# Insights Into Working Alliance: Predictors of Patient‐Perceived Bond With Genetic Counselors Among a Diverse, Multispecialty Sample

**DOI:** 10.1002/jgc4.70308

**Published:** 2026-10-22

**Authors:** Ian M. MacFarlane, Elena R. Fisher, Deborah Cragun, Heather A. Zierhut

**Affiliations:** ^1^ Department of Genetics, Cell Biology, and Development University of Minnesota Minneapolis Minnesota USA; ^2^ Department of Community Health Sciences, College of Public Health University of South Florida Tampa Florida USA

**Keywords:** bond, genetic counseling, patient experiences, therapeutic alliance, working alliance

## Abstract

The Reciprocal Engagement Model of genetic counseling practice centers the patient‐provider relationship as an essential component of practice, but few quantitative studies have assessed what influences this relationship in actual genetic counseling practice. Our study explored which factors are most strongly associated with the patient‐perceived bond subscale of the Working Alliance Inventory in a variety of genetic counseling sessions using a diverse patient sample across multiple clinical specialties. There were 416 participants (50.4% non‐Hispanic white) seen by 41 genetic counselors (95.1% non‐Hispanic white) in a variety of specialty clinics, session modalities, and session types (i.e., pre‐ vs. post‐test) as part of Stage 1 of the GC‐PRO study, which investigated how in‐session processes affect patient‐reported outcomes at two academic institutions and one telehealth company. Participants completed multiple validated scales measuring patient empowerment, satisfaction, decisional conflict, and other patient experience measures (e.g., working alliance and patient‐centered communication). Using hierarchical linear regression modeling, predictors of the bond between participants and their genetic counselors were assessed. There were 6 significant predictors in the final model (*R* ^*2*^_*a*_ = 0.45). Higher patient‐reported bond scores were associated with younger participants, Black participants, more patient‐centered communication, higher patient‐perceived levels of autonomy support, higher post‐session patient‐reported empowerment, and higher session satisfaction levels. Patient autonomy and empowerment had the largest effects and thus may be the most efficient path toward improving bond, but further research is needed to determine causal directionality. Few demographic categories were associated with a diminished bond score, suggesting that patients were generally able to connect with their genetic counselor equally well, though Black patients' stronger bonds merits further exploration.

## Abstract (highlights)

**What Is Known About This Topic:**

- While the relationship between a patient and their genetic counselor is widely considered essential to good practice, there has been little data measuring which factors actually influence this connection in real‐world clinics. Prior research in genetic counseling settings has found that a good working alliance might relate to higher patient empowerment and certain communication components, but these trends have not been tested among large, diverse patient groups and across clinical settings.

**What This Paper Adds to the Topic:**

- This study provides evidence that patient empowerment and support for patient autonomy appear to be strong predictors of a high‐quality patient‐genetic counselor bond. It also contributes valuable new insights by showing that genetic counselors are largely successful at bonding with patients across different backgrounds, while identifying a surprisingly strong therapeutic bond among Black patients that warrants future study.

## Plain Language Summary

- This study surveyed 416 genetic counseling patients to understand which factors are related to a strong personal bond with their genetic counselors. We found that patients reported a stronger bond with their genetic counselor when they felt empowered, satisfied with their genetic counseling session, and actively supported in making their own healthcare choices. While most patients connected equally well regardless of background, Black patients and younger patients reported stronger bonds with their counselors as compared with other patient groups.

## 1 Introduction

In modern medicine, the term working alliance (or therapeutic alliance) is often used to describe ways in which a patient and provider work together to improve health (Doran 2016). Bordin (1979) proposed a three‐component model of working alliance: (1) mutually agreed‐upon goals, (2) a series of tasks to accomplish the goals, and (3) the bond between therapist and client. Working alliance is a dynamic concept that varies across the duration of the relationship and requires both development and maintenance to reach optimal levels in a therapeutic setting (Bordin 1983). Bordin's (1979) model was intended for a psychotherapeutic context, but the goal and bond components largely align with the concepts of contracting and building rapport in genetic counseling, respectively.

Working alliance has been empirically tied to a multitude of patient outcomes in psychotherapy and these relationships are being extended to other healthcare fields, including genetic counseling (e.g., Caleshu et al. 2022; Meystre et al. 2013). Specifically, stronger working alliances are associated with higher levels of shared decision‐making (Sturgiss et al. 2019), patient‐experienced empathy (Hunik et al. 2021), patient‐perceived patient‐centeredness (Hunik et al. 2021), decreases in pain ratings (Holmes et al. 2023), patient satisfaction (Fuertes et al. 2007; Fuertes, Rubinstein, et al. 2017; Fuertes, Toporovsky, et al. 2017; Kim et al. 2008; Toporovsky et al. 2022), and treatment adherence (Fuertes et al. 2007; Fuertes, Rubinstein, et al. 2017; Fuertes, Toporovsky, et al. 2017; Toporovsky et al. 2022). These studies all conceptualize working alliance as a process that facilitates other outcomes, which is how it is most often situated in psychotherapy models. Due to the importance of working alliance in a variety of outcomes, many studies have explored predictors of the alliance itself to better understand how it functions and explore ways to maximize its effect (e.g., Nof et al. 2021; Meier et al. 2005; Renner et al. 2012). Still others have included working alliance as both an outcome and a predictor of various constructs (e.g., Albaum et al. 2020).

### 1.1 Working Alliance in Psychotherapy

A variety of measures exist to quantify the working alliance, with the most common being the Working Alliance Inventory (WAI; Horvath and Greenberg 1989) and its revised short form (WAI‐SR; Hatcher and Gillaspy 2006), which are based on Bordin's (1979) model of the working alliance. WAI measures have parallel forms addressing the working alliance as perceived by the client (WAI‐C), the therapist (WAI‐T), and observers of the clinical interaction (WAI‐O). Numerous meta‐analyses over the past 35 years have consistently found positive effects of working alliance on a variety of therapeutic outcomes (Flückiger et al. 2020; Horvath and Symonds 1991; Martin et al. 2000). There is also evidence that a stronger working alliance can mitigate some of the negative impact of therapists' microaggressions on psychological wellbeing (Owen et al. 2011).

### 1.2 Working Alliance in Genetic Counseling

The centrality of the relationship between a genetic counselor and their patient is highlighted in the field's professional competencies, model of practice, and evidence‐based research. The *Practice‐Based Competencies* for training programs accredited by the Accreditation Council for Genetic Counselors (ACGC) explicitly include “Establish a working alliance with client” (Competency 3b; ACGC 2023). The Reciprocal Engagement Model (REM) of genetic counseling practice centers the relationship element as the hub through which the three other elements interact and lists “Relationship is integral to genetic counseling” as one of the model's five tenets (McCarthy Veach et al. 2007). In their elaboration of the REM, Redlinger‐Grosse et al. (2017) found genetic counselors identified establishing a working alliance as the most prevalent strategy for the REM goal of “counselor and patient establish a bond.” The development of an alliance between the genetic counselor and patient was also identified as an outcome for 7 of the 19 REM goals (Redlinger‐Grosse et al. 2016). The need to establish a strong working alliance is also one of the rationales Moscarello et al. (2025) referenced when discussing the BATHE method as a newer evidence‐based tool for psychosocial assessment in genetic counseling.

As working alliance is a central construct to genetic counseling, researchers need to determine how to measure it in order to support evidence‐based practice and evaluate the effectiveness of genetic counseling research interventions. Erby et al. (2021) adapted the observer form of the WAI (WAI‐O) for genetic counseling sessions in which adults received reproductive carrier results from a research study. The primary change involved item terminology adjustments through replacement of “therapy/therapist” with “genetic counseling/genetic counselor.” Observers were asked to consider the goals of the interaction to include “an exploration of concerns related to the carrier results and a consideration of plans for communicating results within the family” (p. 13). Observers achieved moderate to excellent rates of interrater reliability and Cronbach's alpha ranged from 0.89–0.93 for the three subscales (i.e., tasks, bonds, goals). Validation of the therapist (e.g., genetic counselor) and client self‐report versions of the WAI within the genetic counseling context is ongoing, which is crucial given that the client version of the WAI has been most strongly associated with patient outcomes across the literature (Hatcher and Gillaspy 2006; Horvath and Greenberg 1989; Horvath and Luborsky 1993).

There is a growing body of research examining working alliance within genetic counseling, and existing results are generally consistent with findings from the mental health field. Stronger working alliance was associated with higher empowerment scores in a study of 120 genetic counseling patients (89.7% non‐Hispanic white) with arrhythmogenic cardiomyopathy (Murray et al. 2022). Burnout among a sample of 397 clinical genetic counselors was associated with lower counselor‐reported scores on the bond subscale of the WAI‐T specifically (Caleshu et al. 2022); evaluation of the working alliance from the patient's perspective was not assessed in this study. Finally, Lewis et al. (2026) found that sessions with higher WAI scores more often included mutual agenda setting, attention to patients' emotions, and facilitation of decision‐making among 17 patients (82.4% non‐Hispanic white) seen for cardiovascular genetic counseling. The first two studies explored the role of working alliance as a predictor of other outcomes, while the latter explored predictors of the alliance itself.

To further understand the nature of the working alliance and how it relates to other patient‐ and session‐level variables, it makes sense to first establish potential influences on the alliance measures in genetic counseling among a larger sample and in a broader array of clinical settings than in Lewis et al.'s (2026) study. Additionally, given that relational processes may differ across social groups even when overall clinical outcomes appear similar, there may be value in determining if the working alliance differs across patient demographic groups.

Given the established impacts of medical mistrust in healthcare broadly (e.g., Benkert et al. 2019; Shukla et al. 2025) and genetics/genomics specifically (e.g., Angelo et al. 2024; Buseh et al. 2014; Sheppard et al. 2013), understanding which factors predict counselor‐patient bonds may be uniquely critical in the genetic counseling field. There are emerging psychometric concerns that the goal and task subscales of the WAI may not be distinct (Andrusyna et al. 2001), lending further support to focusing on the bond subscale in particular. Broadbridge et al. (2025) assessed method bias and examined the factor structure of the bond subscale of the WAI‐O in a genetic counseling context, resulting in recommendations for how to reliably measure therapeutic bond in genetic counseling settings. Isolating this specific dimension of working alliance may be valuable for understanding patient‐reported experiences in sensitive healthcare fields such as genetics/genomics.

### 1.3 Current Study

Our study explores predictors of patient‐perceived bond (using the bond subscale of the WAI‐SR‐C) across demographic and session variables as well as across an array of patient‐reported experience measures using a diverse patient sample across multiple genetic counseling specialties. By identifying which factors and patient experience measures predict bond, genetic counselors can better discern how to establish and maintain effective bonds, which in turn may support more positive patient outcomes.

## 2 Methods

The dataset was obtained from the larger Genetic Counseling Processes Result in Outcomes (GC‐PRO) study (Stage 1), which was approved by the University of Minnesota IRB (STUDY000011241). Please see Fisher et al. (2024) for an overview of the full protocol. Only portions of the overall protocol relevant to the data analyzed in the present study are described in this manuscript.

### 2.1 Recruitment and Participant Activities

Patients scheduled for a cancer, cardiology, general genetics, or prenatal/reproductive genetic counseling session were recruited between September 2023 and October 2024 from three sites representing different areas of the United States: two academic health centers (University of Minnesota‐M Health Fairview and University of South Florida‐Tampa General Hospital) and one national telehealth company (Genome Medical Inc.). After consenting, patient participants were asked to complete a pre‐session survey, audio recording of their genetic counseling session, a post‐session survey, and an optional follow‐up interview to further explore findings from the quantitative surveys. Participants received a $30 electronic gift card for completing both surveys and the audio recording, and a $25 gift card for completing the optional interview.

Genetic counselors also consented to participate in the study to allow audio recording of their sessions with enrolled patient participants. Genetic counselors were eligible if they were employed at a participating site, were Board‐certified, and provided services in English or Spanish.

### 2.2 Instruments

Given the one‐time nature of genetic counseling appointments, and to ease the cognitive load on patient participants, items on some scales were converted to Likert‐scales (i.e., “Agree” to “Disagree”) rather than frequency‐based scales. Such changes are denoted below.

The pre‐session survey collected information on the following constructs relevant to the present study.

- Demographic information (e.g., age, current gender identity, household income before taxes, marital/partner status, self‐identified race and ethnicity, and level of education).
- General health literacy: assessed using Chew et al.'s (2008) 3‐item Health Literacy Screening scale. Items were presented on a 5‐point Likert scale, consistent with the original measure.
- Pre‐session empowerment: assessed using the 16‐item version of the Genetic Counseling Outcome Scale (GCOS‐16; Borle et al. 2023). The items were presented on a 5‐point Likert scale (instead of the original 7‐point scale).
- Healthcare decision‐making preference: assessed using a single item from the Cassileth Information Styles Questionnaire (CIS‐Q; Cassileth et al. 1980). Participants were asked if the final decision about healthcare options should be made by themselves, their provider, or a collaborative decision.

The post‐session survey collected information on the following constructs relevant to the present study:

- Bond: assessed by the 4‐item Bond subscale from the Working Alliance Inventory‐Short Revised Form (WAI‐SR; Hatcher and Gillaspy 2006), adapted to genetic counseling by specifying “the genetic counselor” in the place for the therapist name on the original. The items were presented on a 7‐point Likert scale (e.g., “Strongly disagree” to “Strongly agree”) instead of the original 7‐point frequency‐based scale from “Seldom” to “Always” (given the one‐time nature of genetic counseling appointments).
- Autonomy: assessed using the 6‐item Health Care Climate Questionnaire (HCCQ; Czajkowska et al. 2017), which measures patients' perception of their provider as supportive of their autonomy using 7‐point Likert scales.
- Perceived health literacy demand of appointment: assessed using three items adapted from the Consumer Assessment of Healthcare Providers and Systems (CAHPS) Supplemental Health Literacy Item Set (Weidmer et al. 2012) presented on a 7‐point Likert scale.
- Information overload: assessed using five items adapted from Jensen et al.'s (2014) questions about state‐based cancer information overload presented on a 5‐point Likert scale.
- Patient‐centered communication (PCC) was assessed using the 7‐item Patient‐Centered Communication scale (Moser et al. 2022), which measures patients' perceptions of how often providers engaged in core PCC behaviors (i.e., inviting questions, attending to emotions, involving patients in decisions, explaining information clearly, spending sufficient time, supporting self‐management, and helping patients manage uncertainty).
- Patient satisfaction was assessed using the 6‐item Genetic Counseling Satisfaction Scale (GCSS; DeMarco et al. 2004).
- Post‐session empowerment was assessed using the GCOS‐16.
- Overall quality of the genetic counseling visit was assessed by a single item on a 0–10 scale taken from the CG‐CAHPS (Liang et al. 2017).

Study staff additionally collected information about the session itself (e.g., session length, clinical specialty, service modality, pre‐ or posttest counseling, whether the patient presented alone or with a support person).

### 2.3 Data Analysis

Descriptive statistics were calculated for all study variables. We used hierarchical linear regression to determine predictors of bond. To loosely parallel the approach in Flückiger et al. (2020) meta‐analysis that separated predictors into intake characteristics and simultaneous processes, predictors were arranged in four blocks and tested sequentially: (1) demographics; (2) presession scores on patient empowerment and the healthcare decision‐making preferences item; (3) session characteristics; and (4) post‐session assessments via the patient‐reported outcome and experience measures. Notably, Flückiger and colleagues did not incorporate provider‐level variables in their model. As our focus is on patients' perception of bond, we decided to use only variables associated with the patients themselves (Blocks 1 and 2, which Flückiger et al's refer to as intake characteristics), the structure of the session (Block 3), and what happened in the session (Block 4, which Flückiger et al's refer to as simultaneous processes). Significance was assessed at the *p* = 0.05 level. An a priori power analysis determined 190 participants were needed to reach 0.80 power for moderate effect sizes ($\eta_{p}^{2}$ = 0.13; Cohen 1988).

## 3 Results

### 3.1 Participants

The 416 patient participants in this study primarily identified as women (85.9%; see Table 1 for full demographics and session variable descriptives). While the largest single racial/ethnic identity reported was non‐Hispanic white (50.4%), this diverse sample had 16.5% Hispanic, 13.2% Asian, 11% Black, and 8.6% multiracial participants. Most patients had not seen a genetic counselor previously (73.1%) but almost half had previous genetic testing experience (44.8%), whether health‐related testing, paternity testing, ancestry testing, or direct‐to‐consumer genetic screening. The sessions were primarily attended without others (71.9%) and involved pre‐test counseling about the option to pursue genetic testing (79.6%), but the modality was fairly balanced (42% video, 34.1% phone, and 24% in‐person). Sessions were primarily from prenatal (36.9%) and cancer (33.1%), with the remainder coming from cardiology (14.1%) or general genetics (15.6%). The average session length was 37.74 min (SD = 13.63; Mdn = 37). Sessions were primarily conducted in English (98.3%), with only 7 sessions (1.7%) utilizing a Spanish interpreter.

**TABLE 1:** Descriptive statistics for participant demographics, session characteristics, and pre‐session variables.

Personal			Session			Medical		
Variable	*n*	%	Variable	*n*	%	Variable	*n*	%
*Race/Ethnicity*			*Specialty*			*Previously seen a genetic counselor*		
Asian	55	13.2	Cardio	59	14.1	Yes	112	26.9
Black	46	11.0	Cancer	138	33.1	No	304	73.1
Hispanic	69	16.5	Prenatal	154	36.9	*Previous genetic testing*		
White, Non‐Hispanic	210	50.4	General	65	15.6	Yes	187	44.8
Multiracial	36	8.6	*Session modality*			No	229	55.2
*Current gender identity*			Video	175	42.0	*Who should make healthcare decisions*		
Woman	358	85.9	Phone	142	34.1	Me	378	90.6
Man	53	12.7	In‐Person	99	24.0	Collaborative w/Provider	31	8.2
Non‐binary or other gender	5	1.2	*Session type*			Provider	5	1.2
*Partnered*			Pretest	332	79.6			
Yes	297	71.2	Posttest	82	20.4			
No	119	28.8	*Participant attended*					
*Highest education level achieved*			Alone	300	71.9			
No bachelor's degree	135	32.4	With others	116	28.1			
Bachelor's degree	164	39.3						
Graduate degree	117	28.1						
**Continuous variable**	***M***		**SD**		**Mdn**		**Range**	
Age	40.49		13.47		36		18–88	
Visit length (min)	37.74		13.63		37		4–86	

The 41 genetic counselor participants who counseled the patient participants in this study were primarily non‐Hispanic white (95.1%) women (87.8%) who completed their graduate training at 18 unique genetic counseling programs across the United States. The average years of clinical practice were 7.28 years (Mdn = 5 years), though experience levels ranged from 1 to 27 years. The median age of the genetic counselor participants was 31 years.

### 3.2 Predictors of Participant‐Perceived Bond With Their Genetic Counselor

Descriptive statistics for the study measures are presented in Table 2. The hierarchical linear regression started with only demographic variables in the model and successively added pre‐session variables, session characteristics, and post‐session evaluations (see Table 3 for full models). In Model 1, the only significant predictor was a positive effect on bond for Black participants compared to white participants (*p* = 0.006). This was only a small effect ($\eta_{p}^{2}$ = 0.02) and the model only accounted for a small amount of variance in bond scores ($R_{a}^{2}$ = 0.01). The small positive effect for Black participants (*p* = 0.007; $\eta_{p}^{2}$ = 0.02) remained the only significant demographic variable when pre‐session variables were added in Model 2. The only new predictor to reach significance was pre‐session empowerment (*p* < 0.001), which again had a small effect ($\eta_{p}^{2}$ = 0.03) and the model still only predicted a small percentage of the variance ($R_{a}^{2}$ = 0.05). When session characteristics were added in Model 3, the only change to significant predictor was that participants in cancer sessions reported more positive bonds than those in prenatal/reproductive sessions, though the effect was small ($\eta_{p}^{2}$ = 0.01) and the model still poorly predicted overall bond ($R_{a}^{2}$ = 0.08). Notably, session length had no association with bond score.

**TABLE 2:** Descriptive statistics for measures used (*n* = 416).

Scale	*M*	SD	Median	Observed range	Scale range
Overall quality rating of session (CAHPS)	9.41	1.01	10.00	2–10	1–10
Information Overload Scale	1.68	0.57	1.60	1–5	1–5
Working Alliance Inventory—Bond Scale	23.51	3.85	24.00	7–28	7–28
Health Literacy	1.77	0.69	1.67	1–4.67	1–5
Perceived Health Literacy Demand of Appointment	1.69	0.88	1.33	1–6	1–7
Genetic Counseling Outcomes Scale: Pre‐Visit	54.15	5.15	54.00	35–68	16–80
Genetic Counseling Outcomes Scale: Post‐Visit	54.85	5.16	55.00	35–80	16–80
Genetic Counseling Satisfaction Scale	26.21	3.46	26.00	6–30	6–30
Patient‐Centered Communication Scale	26.99	2.77	28.00	8–28	7–28
Healthcare Climate Questionnaire	38.90	4.22	41.00	13–42	6–42

**TABLE 3:** Hierarchical regression model predicting WAI‐bond subscale scores of genetic counseling patients.

Predictor	Model 1^a^			Model 2^b^			Model 3^c^			Model 4^d^		
	*b*	*β*	*p*	*b*	*β*	*p*	*b*	*β*	*p*	*b*	*β*	*p*
Intercept	22.78	—	< 0.001***	16.82	—	< 0.001***	17.52	—	< 0.001***	−7.76	—	0.01*
Asian	0.24	0.02	0.68	0.18	0.02	0.75	0.07	0.01	0.90	0.15	0.01	0.74
Black	1.73	0.14	0.006**	1.67	0.14	0.007**	1.82	0.15	0.003**	0.99	0.08	0.04*
Hispanic	−0.10	−0.01	0.85	0.02	0.00	0.96	0.06	0.01	0.90	−0.15	−0.01	0.72
Multiracial	−0.10	−0.01	0.89	−0.29	−0.02	0.67	−0.17	−0.01	0.81	−0.04	0.00	0.94
Previous genetic counseling session	−0.21	−0.02	0.66	−0.30	−0.03	0.52	−0.19	−0.02	0.68	−0.35	−0.04	0.34
Previous genetic test	0.42	0.05	0.31	0.38	0.05	0.35	0.31	0.04	0.44	0.56	0.07	0.08
Age	−0.02	−0.06	0.20	−0.01	−0.05	0.32	−0.03	−0.10	0.07	−0.03	−0.09	0.04*
Woman	0.89	0.08	0.11	0.91	0.08	0.10	0.62	0.06	0.28	0.86	0.08	0.05
Partnered	0.18	0.02	0.68	0.14	0.02	0.74	0.25	0.03	0.57	0.08	0.01	0.81
No bachelor's	0.31	0.04	0.50	0.36	0.04	0.43	0.28	0.03	0.54	−0.07	−0.01	0.84
Postgraduate degree	0.53	0.06	0.25	0.51	0.06	0.26	0.44	0.05	0.33	0.24	0.03	0.49
General health literacy				−0.31	−0.06	0.26	−0.31	−0.06	0.27	−0.11	−0.02	0.62
GCOS‐16 pre				0.12	0.16	< 0.001***	0.13	0.17	< 0.001***	−0.01	−0.01	0.75
HCP makes decisions				3.21	0.09	0.07	3.24	0.09	0.06	2.19	0.06	0.11
I make decisions				−0.85	−0.09	0.07	−0.64	−0.07	0.17	−0.05	−0.01	0.89
Want all test results				−0.08	−0.01	0.90	0.05	0.00	0.94	−0.24	−0.02	0.64
Cardio							−0.24	−0.02	0.71	0.33	0.03	0.53
Cancer							1.33	0.16	0.02*	0.82	0.10	0.07
General							−0.34	−0.03	0.58	0.14	0.01	0.78
Phone session							−0.99	−0.12	0.08	−0.39	−0.05	0.37
Video session							0.04	0.01	0.94	−0.19	−0.02	0.66
Pre‐test counseling							−0.41	−0.04	0.40	−0.13	−0.01	0.73
Alone in session							−0.35	−0.04	0.48	−0.28	−0.03	0.47
Visit length (min)							0.00	0.02	0.75	0.00	0.01	0.80
Health literacy demands										0.16	0.04	0.45
GCOS‐16 post										0.07	0.10	0.04*
GCSS										0.16	0.14	0.02*
PCC										0.37	0.26	< 0.001***
Healthcare climate										0.34	0.38	< 0.001***
Counselor rating										−0.04	−0.01	0.85
Information overload										0.54	0.08	0.14

*Note:* Reference categories are white racial identity; male or nonbinary gender; single; bachelor's degree; HCP and I share decision making; wanting only actionable test results; prenatal, in‐person, posttest session with another person present.

Abbreviations: GCOS, Genetic Counseling Outcome Scale; GCSS, Genetic Counseling Satisfaction Scale; HCP, healthcare provider; PCC, patient‐centered communication.

* *p* < 0.05.

** *p* < 0.01.

*** *p* < 0.001.

^a^ $R_{a}^{2}$ = 0.01.

^b^ $R_{a}^{2}$ = 0.05.

^c^ $R_{a}^{2}$ = 0.08.

^d^ $R_{a}^{2}$ = 0.45.

The most striking difference between models occurred when post‐session evaluations were added in Model 4. The overall explanatory ability of the model increased dramatically ($R_{a}^{2}$ = 0.45) and while Black participants still displayed a small positive change in bond (*p* = 0.04; $\eta_{p}^{2}$ = 0.01), pre‐session empowerment was no longer significant (*p* = 0.75). Age was a significant predictor only in this final model (*p* = 0.04), with older participants having a small association with lower bond scores ($\eta_{p}^{2}$ = 0.01). The remaining significant predictors were all positive associations and came from the post‐session evaluations. Post‐session empowerment (*p* = 0.04; $\eta_{p}^{2}$ = 0.01) and patient satisfaction (*p* = 0.02; $\eta_{p}^{2}$ = 0.01) had smaller effects similar to those of Black participants and those in cancer sessions, but there were stronger effects on bond for patient perceptions of PCC (*p* < 0.001; $\eta_{p}^{2}$ = 0.07) and autonomy support (*p* < 0.001; $\eta_{p}^{2}$ = 0.08).

## 4 Discussion

Our analysis of the bond between patients and their genetic counselor identified relationships consistent with previous working alliance research in a variety of fields, as well as novel findings. Intentional sampling in our research study permitted the analysis of minoritized racial and ethnic groups which was prioritized in this study to assess whether racial and/or ethnic identity‐based disparities existed in bond as measured by the WAI‐SR‐C. Few demographic variables were predictive of bond in our sample and, when significant, had small effect sizes, the magnitude of which suggests limited practical significance, particularly compared with the stronger associations observed for PCC and patient autonomy support. Even though we cannot conclude strongly that Black participants had a practically meaningful higher bond, these are positive findings in that they suggest genetic counseling patients of various ethnic/racial backgrounds are similarly able to achieve a bond with their genetic counselor. Overall, we did not see prominent differences in bond across ethnic and racial groups.

There is mixed evidence regarding the impact of race on working alliance in other fields. Some studies have found no racial differences in patient‐reported alliance (e.g., Cheng et al. 2023) while others have found lower alliance scores for Black, Indigenous, and/or Persons of Color (BIPOC) patients, specifically those who identify as Black (Eliacin et al. 2018; Li et al. 2024) or Asian and Pacific Islander (Li et al. 2024). Some studies have suggested the concordance between the patient's and provider's racial identities may be more important than the identity per se (e.g., Cheng et al. 2023; Wintersteen et al. 2005). Lukowitsky et al. (2024) findings suggest clinicians' cultural competence (as perceived by patients) may moderate the impact of race on alliance measures. Development of assessments of multicultural counseling skills in genetic counselors would allow us to test this possibility. We could not find any reports of Black patients reporting higher alliance scores than White patients in prior literature and given the demographics of the genetic counselor participants in our sample (e.g., 95.1% white; BIPOC counselors each only had one session with a patient with their same racial identity), racial concordance does not appear to explain our results. Genetic counselors with more pro‐white implicit biases have also been found to provide minoritized simulated patients with less informational individuation and fewer facilitation and activation statements, which resulted in fewer questions being asked and less sharing of psychosocial information by racial/ethnic minoritized patients (Lowe et al. 2020). Further exploration of Black patients' experiences and how the genetic counselor participants in this study approached sessions with Black patients may help us understand why the Black participants in this study showed a very small increase in bond over the white participants despite being racially discordant with their genetic counselor.

Younger patients and those undergoing cancer genetic counseling had higher bond scores in our sample. The negative association between age and bond is not immediately intuitive. With the rapid growth of the field, genetic counselors tend to, on average, come from younger age groups (National Society of Genetic Counselors 2024), which was true for our sample as well (Mdn = 31 years of age). It is possible that age‐related preferences on patient‐prioritized outcomes of genetic counseling may be playing a role in patients' assessments of bond, like the association hypothesized for racial identity (Cheng et al. 2023; Wintersteen et al. 2005), but additional exploration is greatly needed in this area. Similarly, the explanation for patients in cancer genetic counseling sessions reporting higher bond scores is also unclear. De Vries et al. (2017) did find scores even higher than our sample did on the WAI‐SR‐C bond scale (*M* = 25.6) for physicians working in cancer care, but without comparable data from other physician specialties, it is difficult to draw conclusions.

Positive associations with bond for patient satisfaction are consistent with many studies in other healthcare fields (Fuertes et al. 2007; Fuertes, Rubinstein, et al. 2017; Fuertes, Toporovsky, et al. 2017; Kim et al. 2008; Toporovsky et al. 2022). The magnitude of the effect is different, though, as moderate to strong effects are reported for the cited studies whereas our effect was small. Our study did include more predictors than the comparison research, none of which included measures of patient‐centeredness or patient autonomy support, so it may be that satisfaction in those studies was getting credit for variance that was parceled out in our study. With more variables in our study, we may be seeing a more accurate level of the impact of satisfaction. Alternatively, our study might be diluting the effect of satisfaction by splitting it across constructs that are overly similar. Future studies could look to replicate findings from other fields more directly to compare effect sizes.

Patient empowerment and patient autonomy support similarly demonstrated positive association patterns with counselor‐patient bond. Murray et al. (2022) demonstrated a positive association of working alliance with patient empowerment in the cardiogenetics space, but our cross‐specialty and racially/ethnically diverse sample only showed a small effect while they had a moderate one. Murray et al. were using the change in empowerment from pre‐ to post‐session while we used the raw scores, so this may account for at least part of the difference. The effect of patient‐centeredness in a study of patients seeking care in general practice settings in Australia was moderate‐sized (Hunik et al. 2021), which was again consistent in direction but stronger than the small effect observed in our genetic counseling study. The same caveats apply in terms of our higher number of variables and our inclusion of patient autonomy support, which was not included in the Hunik et al. (2021) study. Overall, our results generally support the construct validity of the WAI‐SR‐C for genetic counseling practice, in that the pattern of associations with other patient experience measures is fairly consistent with those seen in other fields.

Contrary to prior observer‐rated work in genetic counseling (Erby et al. 2021), session length was not significantly associated with patient‐reported bond in the present study. This finding suggests that the amount of time spent in a genetic counseling session may not, by itself, determine whether patients experience a strong bond with the genetic counselor. Although longer sessions may provide more opportunities for observable bond‐building behaviors, such as agenda setting, clarification of goals, emotional responsiveness, and shared decision‐making, patients may evaluate bond more globally, based on whether they felt heard, respected, supported, and involved in the session (Pérez‐Rojas et al. 2019). Thus, patient‐reported bond may depend less on session duration than on the patients' perceived quality of the interaction, which in this study was captured by the PCC and autonomy support measures that explained the most variation in scores measuring the patients' perceptions of working alliance bond.

Finally, it is also notable that health literacy was not a significant predictor in our models. Health literacy and patient‐reported bond do not appear to be commonly paired in the research literature, but Kamimura et al. (2020) found a positive effect of health literacy on patients' assessment of the patient‐provider relationship. The conflicting findings may be due to the difference in relationship measures (WAI vs. Health Relationship Trust scale, Bova et al. 2006; and a communication and partnership with providers scale, Little et al. 2001) and patient populations (genetic counseling vs. primary care patients), but additional exploration is warranted. The impact of health literacy on patient‐physician communication is more established, as a recent scoping review found health literacy was one of the most common barriers (when low) and facilitators (when high) across studies that included both patient and provider participants (Vamos et al. 2024). Health literacy is also entwined with empowerment (Schulz and Nakamoto 2013), so modeling more complex relationships via interactions and/or indirect effects in future studies may offer a clearer picture. Our findings, however, suggest that lower health literacy did not impede bond formation in genetic counseling settings.

### 4.1 Strengths and Limitations

The ability to test racial/ethnic differences, multiple modalities, and multiple clinical specialties due to the diversity of the GC‐PRO dataset was a strength of the study. While our sample does not represent the full diversity of genetic counseling patients, counselors, or sessions, we did intentionally over‐sample for BIPOC patients in this study to ensure findings were as representative as possible of patient populations across the United States.

The final model showed strong explanatory power, adjusted *R* ^2^ = 0.45, indicating that the included variables accounted for just under half of the variance in WAI bond scores. However, most of this explanatory gain occurred after adding patient experience constructs measured post‐session, which are conceptually and temporally close to WAI bond. Analysis of follow‐up interviews with patients debriefing their sessions may uncover additional variables to explore that may explain additional variance in WAI bond scores. Some of the missing variance may also be accounted for in provider‐level variables (e.g., years of experience), so these should be explored in future studies. We also did not include interactions in the model, which may have identified more nuanced relationships, particularly between identity variables to account for the intersectionality of multiply‐marginalized populations.

Patient surveys indicated that the genetic counselors would not be shown the results of the survey, but it is possible participants did not want to critique the genetic counseling session regardless of this disclaimer. There may also be a potential selection effect of those who declined to participate in the GC‐PRO study due to negative expectations; however, the fact that consent to participate was secured independently of and prior to the interaction with the genetic counselor limits the impact of selection effects based on how the session itself went. Additionally, our high post‐visit survey completion rate (95%) suggests that those who did have a negative experience with their genetic counseling sessions may still be captured within the dataset.

Lastly, our bond scores had substantive ceiling effects, with 50% of scores within approximately 1 standard deviation of the top of the scale. This attenuated range limits the predictive ability of the model and may obscure relationships among study variables, though the widespread ceiling effects across working alliance measures (e.g., Meier and Feeley 2022) at least means our models are still comparable with those in other fields. It is also unclear what effect changing the rating scales may have on how patients interpreted items on these scales, so further psychometric exploration is needed. Finally, since working alliance development is likely happening concurrently with other patient changes such as patient empowerment, it is unclear if it is best modeled as a predictor or an outcome. As our data were observational, we cannot prove causal directionality.

### 4.2 Research Recommendations

The mixed‐methods, multisite, multispecialty dataset from Stage 1 of the GC‐PRO study is fertile ground for exploration of numerous aspects of genetic counseling sessions and patient‐reported outcomes. Additional analyses from the GC‐PRO dataset will continue to assess the psychometrics of the WAI‐SR as well as working alliance itself as a predictor of patient outcomes. Many of our significant predictors had small effect sizes, so determining whether these impacts are clinically meaningful requires additional research. Interviews regarding key moments in sessions may reveal associations not reflected in the quantitative data. Using methods like interpersonal process recall (Larsen et al. 2008) could help tie even more specific events or counseling processes/techniques in sessions to the development of bond as well as fluctuations over time. Comparing patient, genetic counselor, and observer ratings of the same session would provide valuable insight into how perceptions align or not, which may have implications for contextualizing outcomes in genetic counseling like it does in psychotherapy (Marmarosh and Kivlighan Jr 2012).

More research continues to be needed across different outcomes beyond bond and in larger numbers of diverse patient populations. A review found few studies that compared genetic counseling outcomes across racial groups (Southwick et al. 2020), so research in this area should continue to be a priority for the field. If the current findings related to bond do replicate, this would be an encouraging foothold toward improving equitable experiences and creating inroads to improve uptake of services.

### 4.3 Practice Recommendations

Even with a robust sample, practice recommendations resulting from this analysis should be cautiously interpreted until the findings replicate. Our findings do suggest that emphasis on patients' feelings of autonomy support and PCC practices may be key to developing stronger bonds with patients. Emphasizing assessment of patient goals during contracting and shifting priorities to align with patients' evolving needs during sessions may enhance feelings of autonomy support, thereby strengthening the potential for a higher bond between the counselor and patient. Similarly, engaging in core PCC behaviors (i.e., inviting questions, attending to emotions, involving patients in decisions, explaining information clearly, spending sufficient time, supporting self‐management, and helping patients manage uncertainty) may also help to lay the groundwork for developing stronger bonds with patients.

## 5 Conclusions

This study is one of the largest explorations of patient‐perceived bond in the genetic counseling field to date and sheds important light on predictors of this crucial relationship between patients and genetic counseling providers. The significant predictors identified in this study (i.e., younger participants, Black participants, more PCC, higher patient‐perceived levels of autonomy support, higher post‐session patient‐reported empowerment, and higher session satisfaction levels) are largely consistent with preexisting literature in other fields, providing evidence toward construct validity of the measure in genetic counseling and encouraging further explorations to distinguish which findings translate from other fields and which factors might be unique to genetic counseling. The limited impact of demographic variables including race and ethnicity is a positive finding that suggests bond with genetic counselors is not dependent on patient identity, though intersectional identity statuses still need to be explored.

## Author Contributions

**Ian M. MacFarlane:** conceptualization, investigation, writing – original draft, writing – review and editing, methodology, formal analysis, supervision. **Elena R. Fisher:** writing – original draft, writing – review and editing, data curation, project administration. **Deborah Cragun:** conceptualization, writing – review and editing, supervision, funding acquisition, data curation. **Heather A. Zierhut:** funding acquisition, writing – review and editing, conceptualization, project administration, supervision, data curation.

## Funding

This work was supported by the National Human Genome Research Institute, R01HG011916 and the National Center for Advancing Translational Sciences, UL1TR002494 (University of Minnesota). This content is solely the responsibility of the authors and does not necessarily represent the official views of the National Institutes of Health.

## Disclosure

AI statement: No artificial intelligence tools were used for the research project or to develop this manuscript.

## Ethics Statement

This study was approved by the University of Minnesota Institutional Review Board on November 30, 2022 (STUDY00011241). The research team followed standard ethical research standards.

## Consent

All participants gave their informed consent prior to their inclusion in the study.

## Conflicts of Interest

The authors declare no conflicts of interest.

## Data Availability

The data that support the findings of this study are openly available in Open Science Framework at https://doi.org/10.17605/OSF.IO/ZATUQ.
